# Migration/Differentiation-Associated LncRNA *SENCR* rs12420823*C/T: A Novel Gene Variant Can Predict Survival and Recurrence in Patients with Breast Cancer

**DOI:** 10.3390/genes13111996

**Published:** 2022-10-31

**Authors:** Essam Al Ageeli, Samy M. Attallah, Marwa Hussein Mohamed, Amany I. Almars, Shahad W. Kattan, Eman A. Toraih, Manal S. Fawzy, Marwa K. Darwish

**Affiliations:** 1 Department of Clinical Biochemistry (Medical Genetics), Faculty of Medicine, Jazan University, Jazan 45142, Saudi Arabia; 2 Department of Clinical Pathology, Faculty of Medicine, Mansoura University, Mansoura 35516, Egypt; 3 Department of Clinical Pathology, King Fahad Armed Forces Hospital, Jeddah 23311, Saudi Arabia; 4 Department of Medical Biochemistry and Molecular Biology, Faculty of Medicine, Suez Canal University, Ismailia 41522, Egypt; 5 Department of Medical Laboratory Sciences, Faculty of Applied Medical Sciences, King Abdulaziz University, Jeddah 21589, Saudi Arabia; 6 Department of Medical Laboratory, College of Applied Medical Sciences, Taibah University, Yanbu 46411, Saudi Arabia; 7 Division of Endocrine and Oncologic Surgery, Department of Surgery, Tulane University School of Medicine, New Orleans, LA 70112, USA; 8 Genetics Unit, Department of Histology and Cell Biology, Suez Canal University, Ismailia 41522, Egypt; 9 Department of Biochemistry, Faculty of Medicine, Northern Border University, Arar 1321, Saudi Arabia; 10 Chemistry Department (Biochemistry Branch), Faculty of Science, Suez University, Ismailia 41522, Egypt; 11 Department of Medical Laboratories Sciences, College of Applied Medical Sciences, Shaqra University, Al-Quwaiiyah 19257, Saudi Arabia

**Keywords:** breast cancer, real-time PCR, recurrence, rs12420823, *SENCR*, single nucleotide polymorphism, survival

## Abstract

Long non-coding RNAs (lncRNAs) have key roles in tumor development and the progress of many cancers, including breast cancer (BC). This study aimed to explore for the first time the association of the migration/differentiation-associated lncRNA *SENCR* rs12420823C/T variant with BC risk and prognosis. Genotyping was carried out for 203 participants (110 patients and 93 controls) using the TaqMan allelic discrimination technique. The corresponding clinicopathological data, including the recurrence/survival times, were analyzed with the different genotypes. After adjustment by age and risk factors, the T/T genotype carrier patients were more likely to develop BC under homozygote comparison (T/T vs. C/C: OR = 8.33, 95% CI = 2.44–25.0, *p* = 0.001), dominant (T/T-C/T vs. C/C: OR = 3.70, 95% CI = 1.72–8.33, *p* = 0.027), and recessive (T/T vs. C/T-C/C: OR = 2.17, 95% CI = 1.08–4.55, *p* < 0.001) models. Multivariate logistic regression analysis showed that the T/T genotype carriers were more likely to be triple-negative sub-type (OR = 2.66, 95% CI = 1.02–6.95, *p* = 0.046), at a higher risk of recurrence (OR = 3.57, 95% CI = 1.33–9.59, *p* = 0.012), and had short survival times (OR = 3.9, 95% CI = 1.52–10.05, *p* = 0.005). Moreover, Cox regression analysis supported their twofold increased risk of recurrence (HR = 2.14, 95% CI = 1.27–3.59, *p* = 0.004). Furthermore, the predictive nomogram confirmed the high weight for *SENCR* rs12420823*T/T and C/T genotypes in predicting recurrence within the first year. The Kaplan–Meier survival curve demonstrated low disease-free survival (T/T: 12.5 ± 1.16 months and C/T: 15.9 ± 0.86 months versus C/C: 22.3 ± 0.61 months, *p* < 0.001). In conclusion, the LncRNA SENCR rs12420823*C/T may be associated with an increased risk of BC in women and could be a promising genetic variant for predicting recurrence and survival.

## 1. Introduction

Breast cancer (BC) is the leading cancer that impacts women in terms of incidence/morbidity, with an estimated rate of 268,600 new cases per year [1]. Better prognosis is still related to early-stage detection, emphasizing timely and improved screening strategies [2]. Since many molecular mechanisms could influence BC onset and progress, unraveling the molecular players in these biological functions and cellular alterations could help in early risk assessment, prognostication at the time of diagnosis, and future targeted therapy [3,4].

Accumulating evidence has realized that more than 90% of the total genome is actively transcribed, but not all DNA sequences have the protein-coding potential [5]. Non-coding RNAs (ncRNAs) are of increasing interest as their genetic variants and expressions are often altered in various malignancies, including BC [6,7,8,9,10,11,12].

One family of ncRNAs, long non-coding RNAs (lncRNAs; >200 bp), have been implicated in BC tumorigenesis and progression via multiple genetic/epigenetic mechanisms in a tissue-specific pattern [12]. Several genetic variants are also associated with deregulated expression of these lncRNAs in BC [13,14,15,16,17,18].

The “Smooth muscle and endothelial cell-enriched migration/differentiation-associated lncRNA” SENCR, also known as “FLI1 Antisense RNA 1,” is a newly discovered lncRNA encoded by a gene (ID: 100507392) on chromosome 11q24.3 (https://www.ncbi.nlm.nih.gov/gene/100507392) (accessed on 2 September 2022). This human-specific, vascular cell-enriched lncRNA was shown to be upregulated in human coronary artery smooth muscle cells and to be involved in the maintenance of membrane homeostasis in vascular endothelial cells [19]. Its knockdown significantly affected the expression of several genes related to smooth muscle cell contraction and cell migration [20].

Our prior studies showed the genetic variant rs12420823 C/T (chr11: 128693497) to be common in our population [21,22]. Since genetic alterations, including single nucleotide polymorphisms (SNPs), can influence cancer susceptibility/risk and may have predictive/prognostic value in cancer, the authors were interested in exploring for the first time the association of rs12420823 C/T SNP with BC risk and/or prognosis.

## 2. Materials and Methods

### 2.1. Study Subjects

The present study enrolled 110 women with histologically confirmed primary BC who attended the Oncology Diagnostic Unit, Suez Canal University, Ismailia, Egypt, for histopathological and follow-up studies from January 2017 to August 2019, and 93 cancer-free women matched the patients in age, residency, and time of sample collection. All participants had no history of chronic diseases, including inflammatory and autoimmune disorders, and the patient group did not receive any treatment modalities before blood sampling. The demographic and clinical data of patients with BC were obtained from the patients’ medical records. The breast cancer pathological grade was evaluated according to “Elston and Ellis modification of Scarff-Bloom-Richardson classification” [23], and the clinical stage was specified following the “American Joint Committee on Cancer (AJCC) tumor–node–metastasis (TNM) classification system” [24]. The receptor expression status of the cancer tissues “(estrogen receptor; ER, progesterone receptor; PR, and the human epidermal growth factor receptor 2; HER2)” was retrieved from the medical records. The patient’s prognosis was evaluated by “Nottingham Prognostic Index (NPI)” and “Immunohistochemical Prognostic Index (IHPI)”. At the same time, the predicted risk of recurrence for each patient was calculated according to the “European Society of Medical Oncology (ESMO)” clinical recommendations for follow-ups of primary BC [6].

The authors followed the “Declaration of Helsinki’s ethical guidelines”. The local “Medical Research Ethics Committee” of the Faculty of Medicine, Suez Canal University, approved the present study. Written consent was taken from all participants before taking part.

### 2.2. SENCR Mutation in Cancer Databases

The mutation spectrum of the *SENCR* gene in cancer was identified from The Cancer Genome Atlas (TCGA, https://cancergenome.nih.gov/, accessed on 20 August 2022), which contains genome maps from >30 cancer types. The role of the *SENCR* gene in cancer hallmarks was determined using the Cancer Hallmarks Analytics Tool (CHAT) (http://chat.lionproject.net/, accessed on 20 August 2022), a web browser for classifying cancer-related texts and text mining associated biological processes from PubMed articles [25]. The prognostic value of the *SENCR* gene in patients with BC was explored in Kaplan–Meier Plotter (https://kmplot.com/analysis/, accessed on 20 August 2022), which includes gene chip and RNA-seq data from microarray and TCGA data [26]. 

### 2.3. Selection of SENCR Gene Variant

The SENCR gene maps to chromosome 11q24.1 (Chromosome 11: 128,691,664–128,696,023 reverse strand), which overlaps the 5-prime end of the Friend Leukemia Integration 1 (FLI1) Proto-Oncogene Transcription Factor gene in the antisense direction. The SENCR gene contains three exons and spans approximately 2 kb. It encodes three splice variant transcripts, namely SENCR-201 of 1298 bp, SENCR-202 of 534 bp, and SENCR-203 of 425 bp. In the Ensembl Genomic Database, the SENCR gene contains 2633 intron variants and 559 non-coding transcript exon variants. Of these, the most common mutation was rs12420823 C/T (chr11: 128,693,497) which covers an intron of two transcripts of SENCR in the negative strand (n.112-106G > A and n.77-1376G > A) and five transcripts of the FLI1 gene in the positive strand. The SNP was selected for genotyping in association with breast cancer.

### 2.4. SENCR rs12420823*C/T Allelic Discrimination Analysis

Genomic DNA was isolated from peripheral blood (3 mL) collected in EDTA vacutainers using “QIAamp DNA Blood Mini kit (Cat. No. 51104, QIAGEN, Hilden, Germany)” following the manufacturer’s protocols. The extracted DNA concentration and purity were assessed by a “Nanodrop-1000 spectrophotometer (NanoDrop Tech., Wilmington, NC, USA)”. The isolated DNA samples were stored at −80 °C until the molecular work (allelic discrimination polymerase chain reaction (PCR) analysis). The rs12420823*C/T transition substitution genotyping was run using a functionally validated TaqMan allelic discrimination assay guided by the manufacturer’s instructions. The assay “(C__11783392_10, Catalog #: 4351379, Applied Biosystems, Foster City, CA, USA)” contains specified probes to determine the wild/mutant alleles in the context sequence: “[VIC/FAM]GGCGCTGGGTTACCCGCAGCCCTAG[C/T]CAACTCTCCCTCCATACCC CCCCTA] according to the build GRCh38.” The component type and concentrations of each PCR run were detailed previously [27]. The PCR was carried out blindly to the case/control status of the samples by two coauthors on a “StepOne™ Real-Time PCR System (Applied Biosystems)”. Each PCR run started with initial denaturation for 10 min at 95 °C, followed by 40 amplification cycles for 15 s at 95 °C/annealing for 1 min at 60 °C, then a final step for 30 s at 60 °C [28]. Negative controls were tested with each run to rule out carryover contamination, and about ten percent of the total samples were randomly reanalyzed in a separate run with a concordance rate of 100%. Post-PCR data analysis was carried out by SDS software (v1.3.1., Applied Biosystems, Foster City, CA, USA).

### 2.5. Statistical Analysis

Data were analyzed using SPSS version 27.0 (IBM Corp. Armonk, NY, USA). Allele and genotype frequencies were calculated as previously described [29]. A Chi-square test was used for comparison. The Hardy–Weinberg equilibrium was estimated using the Online Encyclopedia for Genetic Epidemiology (OEGE) software (http://www.oege.org/software/hwe-mr-calc.shtml) (accessed 20 August 2022). Adjusted odds ratios (OR) with a 95% confidence interval (CI) using logistic regression models were calculated for multiple genetic association models [30]. Akaike information criterion (AIC) was used to select the best model. Association of the SNP with clinical and pathological markers was performed using Fisher’s Exact and Kruskal–Wallis tests. Multivariate Cox regression analysis was implemented, and hazard ratio (HR) with 95% CI was estimated. Kaplan–Meier plot was generated for the genotypes. A two-tailed *p*-value of 0.05 was considered statistically significant.

## 3. Results

### 3.1. Baseline Characteristics of the Study Population

A total of 203 participants (110 patients and 93 controls) were included in this study. There were no significant differences between the two study groups regarding age, residency, and menopausal status (Table 1). A higher frequency of BC cases was observed in the cohort of single patients with a history of breast diseases, early menarche, sedentary lifestyle, and increased body weight than in their counterparts.

### 3.2. Genotype and Allele Frequencies of SENCR rs12420823*C/T Polymorphism

Genotype frequency in controls followed the Hardy–Weinberg equilibrium (*p* = 0.12). Their minor allele frequency (T allele) was 0.44. In comparing the patient group with the control group, the T allele was more frequent in BC women (59.6% versus 44%, *p* < 0.001). Similarly, T/T was the most common genotype among patients (29.1% vs. 15%), while C/C homozygosity was more represented in the control groups (28%) compared to the cancer cohort (10%) (*p* = 0.001) (Table 2).

### 3.3. Association of SENCR rs12420823*C/T Polymorphism with Breast Cancer Risk

Genetic association model analysis showed women with the T variant had a higher risk of breast cancer under homozygote comparison (T/T vs. C/C: OR = 5.26, 95% CI = 2.08–14.3, *p* = 0.001), the dominant model (T/T-C/T vs. C/C: OR = 3.45, 95% CI = 1.61–7.69, *p* = 0.016), and the recessive model (T/T vs. C/T-C/C: OR = 2.33, 95% CI = 1.15–4.76, *p* < 0.001). After adjustment by age and risk factors (Table 3), T/T genotype carriers were more likely to develop breast cancer under homozygote comparison (T/T vs. C/C: OR = 8.33, 95% CI = 2.44–25.0, *p* = 0.001), the dominant model (T/T-C/T vs. C/C: OR = 3.70, 95% CI = 1.72–8.33, *p* = 0.027), and the recessive model (T/T vs. C/T-C/C: OR = 2.17, 95% CI = 1.08–4.55, *p* < 0.001).

### 3.4. Association of SENCR rs12420823*C/T Polymorphism and the Histopathological Types of Breast Cancer

The studied variant was not associated with the histopathological types of BC in the patient group (Table 4).

### 3.5. Association of SENCR rs12420823*C/T with Polymorphism and Risk Factors

The studied variant was not associated with any risk factors in the patient group (Table 5).

### 3.6. SENCR Polymorphism as a Prognostic Marker

Table 6 showed a univariate association between the *SENCR* rs12420823*C/T genotypes and clinicopathological parameters. The rs12420823 T/T genotype carriers were less likely to be positive for estrogen and progesterone receptors (*p* = 0.036) and they had a three times higher risk of recurrence (*p* = 0.006) and shorter survival times (<12 months) (*p* = 0.005) than C/C-C/T genotype carriers.

**Figure 1:**
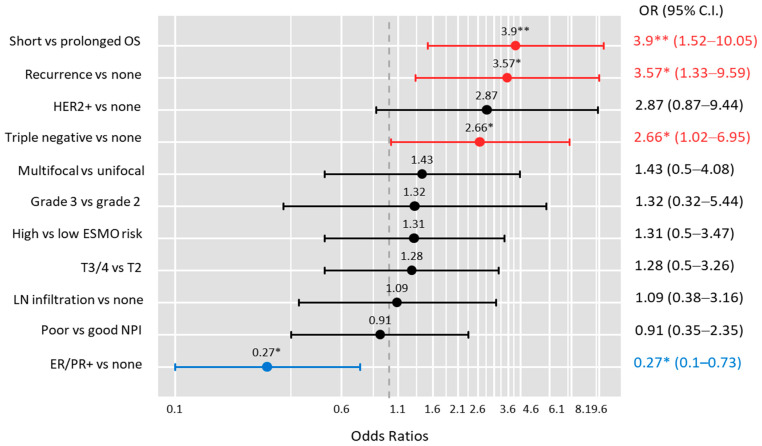
Impact of *SENCR* genotype on poor prognostic markers. Each horizontal bar represents the odds ratio (OR) and confidence interval (CI) of T/T versus C/T-C/C genotype for a single poor outcome in the left panel. The red bar indicates a significant risk (OR > 1.0) and the blue bar indicates a significant protective OR (<1.0). OS: overall survival; ESMO: European Society of Medical Oncology; LN: lymph node; NPI: Nottingham Prognostic Index; ER/PR+: estrogen- and progesterone-positive receptors. * *p* < 0.05, ** *p* < 0.01.

After adjustment by age and risk factors, multivariate logistic regression analysis showed that carriers of the T/T genotype were more likely to have triple-negative receptors (OR = 2.66, 95% CI = 1.02–6.95, *p* = 0.046). Carriers of T/T homozygotes were at a higher risk of recurrence (OR = 3.57, 95% CI = 1.33–9.59, *p* = 0.012) and shorter survival times (OR = 3.9, 95% CI = 1.52–10.05, *p* = 0.005) (Figure 1). In contrast, the C/T–C/C genotype was associated with being 3.7 times more ER/PR-receptor positive (OR = 3.72, 95%CI = 1.36–10.1, *p* = 0.010).

### 3.7. SENCR rs12420823*C/T Polymorphism as a Predictive Marker

**Figure 2:**
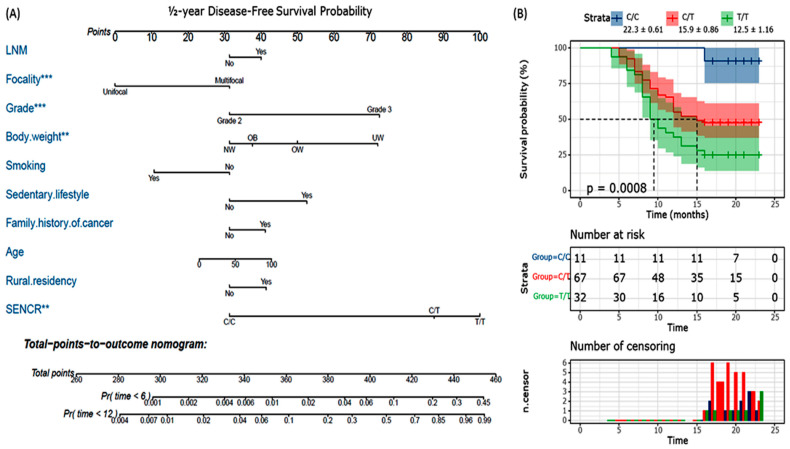
(**A**) Predictive nomogram for breast cancer recurrence. The Cox regression model was used to generate the nomogram. (**B**) Kaplan–Meier curve for disease-free survival analysis. Log-rank test was used to compare the survival probability across *SENCR* rs12420823*C/T genotypes. LNM: lymph, node, metastasis. ** *p* < 0.01, *** *p* < 0.001.

The Cox regression analysis showed that those with the T/T genotype had two times greater risk of recurrence (HR = 2.14, 95%CI = 1.27–3.59, *p* = 0.004). As depicted in Figure 2A, the predictive nomogram showed a high weight for *SENCR* rs12420823*T/T and C/T genotypes predicting recurrence within the first year. The Kaplan–Meier survival curve demonstrated low disease-free survival (T/T: 12.5 ± 1.16 months and C/T: 15.9 ± 0.86 months versus C/C: 22.3 ± 0.61 months, *p* < 0.001).

## 4. Discussion

Breast cancer represents the most prevalent cancer identified among females worldwide. Although it is a treatable disease upon early detection, the incidence of metastasis and resistance to chemotherapy are among the main obstacles to therapy [31]. In this sense, identifying new prognostic genetic markers could improve prognosis and survival.

The importance of lncRNAs has been revealed through their ability to act as promoters of tumorigenesis besides tumor suppressors, providing mounting evidence of their involvement in cancer initiation, progression, and outcomes through several mechanisms [32,33,34,35]. Accumulating evidence supports the association of lncRNA variants with the risk and prognosis of various cancers, including BC [36,37,38]. For example, Bayram et al. suggested that the lncRNA *HOTAIR* rs920778 CC genotype might have a role in genetic susceptibility to BC tumorigenesis and aggressiveness in a “Turkish population” [39]. Lin et al. reported that the “maternally expressed imprinted” *H19* rs217727*T variant could contribute to the risk of BC in a “Southeast China Han population” [15]. Similarly, Cui et al. confirmed the link between *H19* rs2071095 and BC risk [16]. Peng et al. identified that *MALAT1* rs3200401 and rs619586 tag SNPs were associated with BC susceptibility through mRNA expression level dysregulation [40]. Additionally, several studies could unravel the association of lncRNA variants with one or more BC prognostic indices; for instance, Riaz et al. found that *H19* rs2107425 was significantly associated with shorter metastasis-free survival [41]. Moreover, Royds and colleagues identified that *ANRIL* rs11515 was associated with aggressive BC with *ANRIL* gene upregulation and *p16^(INK4a)^* downregulation [42].

The current study analyzed the possible association of the lncRNA *SENCR* rs12420823 variant with breast cancer risk and prognosis. The results revealed that the T/T genotype was significantly associated with a higher risk of BC under homozygote comparison and both the dominant and the recessive models. Although the studied genotypes were not associated with any of the BC risk factors, the T/T genotype carriers had some poor prognostic indices in terms of being less likely to be positive for estrogen and progesterone receptors, having a three times higher risk of recurrence, and shorter survival times (<12 months) than other genotypes’ carriers. Furthermore, the predictive nomogram showed a high weight for *SENCR* rs12420823*T/T and C/T genotypes in predicting recurrence within the first year. To our knowledge, this is the first study reporting a possible association between the lncRNA *SENCR* rs12420823 variant and BC risk/prognosis.

The antisense lncRNA SENCR is a vascular-enriched lncRNA transcribed from the opposite strand of the *FLI1* gene, a member of the E26 transformation-specific (ETS) family [43], which has been described as an early regulator of hematoendothelial development, stimulating angiogenesis of endothelial cells [44] that is crucial for solid tumor development [45]. Moreover, SENCR has been reported to have a promigratory effect that can impact cell cycle progression by stimulating cells to enter the S/G2 phase [44]. Recently, it was reported to be upregulated in cisplatin-resistant non-small cell lung cancer, and its knockdown inhibited cancer cell growth/cisplatin resistance by downregulating *FLI1* expression [46].

On searching “DIANA-LncBase v3” (https://diana.e-ce.uth.gr/lncbasev3/interactions) (accessed on 24 October 2022), which is a repository with experimentally validated miRNA targets on non-coding transcripts [47,48], revealed that SENCR could interact with a high confidence level and sponge the following microRNAs (miRs): homo sapiens (hsa)-let-7b-5p, hsa-let-7c-5p, hsa-miR-122-5p, hsa-miR-155-5p, hsa-miR-210-3p, hsa-miR-342-5p, hsa-miR-629-5p, and hsa-miR-92a-2-5p [49,50]. Interestingly, these sponged microRNAs were reported to be implicated in breast cancer tumorigenesis and/or progression [51,52,53,54,55,56].

The intronic rs12420823*C/T variant is located within a regulatory region of the *SENCR* gene. Its minor allele frequency among the present cohort was 0.44 compared to different populations (Table 7). This variant is likely to modulate the transcription factors (TFs) binding to the gene region as predicted by “HaploReg v4.1” (https://pubs.broadinstitute.org/mammals/haploreg/haploreg.php) (last accessed on 20 September 2022), resulting in potential “T” allele-specific dysregulated expression of the lncRNA SENCR (Table 7).

The validated online prediction tool showed that the studied variant could influence binding with the transcriptional factors “11-zinc finger protein CTCF” and the “CJUN”. Interestingly, the CTCF itself has been implicated in affecting the “estrogen receptor α1; ESR1,” which is a crucial factor in both normal breast development and BC [57], and the CJUN has been associated with cell proliferation, angiogenesis, cell cycle progression, and lower survival rate [58,59]. The possible role of other linked variant(s) also should be confirmed; *SENCR* rs12420823 was found to be in high (r^2^ > 0.80) linkage disequilibrium (LD) with the intronic rs12420835 variant, which has been associated with alteration in nine DNA regulatory motifs. In line with these findings, several studies have confirmed that “trait-associated SNPs” are concentrated in regulatory domains and can perturb transcription factor recognition in these regions, thus conferring “allele-specific dysregulation of the SNP-associated gene” [16,60]. Collectively, these findings could support, in part, the potential association of the studied gene variant with BC risk and poor prognosis; however, the underlying mechanism(s) remain to be elucidated.

Although the authors investigated the association of the studied variant with other disorders such as diabetic retinopathy [21] and coronary artery disease in patients undergoing coronary angiography [22] with no detected associations with disease risk specified, no further published work, up to the current time, has been identified to associate this variant with other disorders, including cancers.

It is worth noting that BC is a complex disorder with multiple “gene-environment” interactions. Exploring one gene/SNP at a time may not be able to identify the modest impact associated with each risky variant. In this sense, taking a “multigenic approach” to identify the impact of several genetic variations as predictors of cancer risk is crucial.

## 5. Conclusions

In the present study, it was shown that *SENCR* rs12420823 SNP could be associated with breast cancer risk and poor prognosis in terms of a higher risk of recurrence and shorter survival times. As this is the first report that unleashes this association as a single-center experience, further large-scale multicenter studies are recommended with functional experiments to elucidate the precise mechanism behind the implication of the studied variant in breast cancer.

## Figures and Tables

**Table 1:** Baseline characteristics and risk factors in the study population.

Characteristics	Levels	Controls (*n* = 93)	Patients (*n* = 110)	*p*-Value
Demographics				
Age	Adolescents (≤21 years)	27 (29)	22 (20)	0.14
	Adults (>21 years)	66 (71)	88 (80)	
Residency	Urban	47 (50.5)	67 (60.9)	0.15
	Rural	46 (49.5)	43 (39.1)	
Marital status	Divorced	20 (21.5)	19 (17.3)	**0.017**
	Married	60 (64.5)	57 (51.8)	
	Single	13 (14)	34 (30.9)	
Menopausal status	Pre-menopause	69 (74.2)	79 (71.8)	0.75
	Post-menopause	24 (25.8)	31 (28.2)	
Risk factors				
Family history of cancer	Negative	73 (78.5)	78 (70.9)	0.25
	Positive	20 (21.5)	32 (29.1)	
Prior breast disease	Negative	93 (100)	100 (90.9)	**0.002**
	Positive	0 (0)	10 (9.1)	
Oral contraceptive pills	Negative	76 (81.7)	89 (80.9)	0.88
	Positive	17 (18.3)	21 (19.1)	
Early menarche	Negative	64 (68.8)	38 (34.5)	**<0.001**
	Positive	29 (31.2)	72 (65.5)	
Nullipara	Negative	76 (81.7)	94 (85.5)	0.56
	Positive	17 (18.3)	16 (14.5)	
Late first gravida	Negative	88 (94.6)	106 (96.4)	0.73
	Positive	5 (5.4)	4 (3.6)	
Late menopause	Negative	83 (89.2)	98 (89.1)	0.97
	Positive	10 (10.8)	12 (10.9)	
No breastfeeding	Negative	81 (87.1)	90 (81.8)	0.33
	Positive	12 (12.9)	20 (18.2)	
Night light exposure	Negative	69 (74.2)	98 (89.1)	**0.009**
	Positive	24 (25.8)	12 (10.9)	
Sedentary lifestyle	Negative	24 (25.8)	11 (10)	**0.005**
	Positive	69 (74.2)	99 (90)	
Smoking	Negative	88 (94.6)	98 (89.1)	0.21
	Positive	5 (5.4)	12 (10.9)	
Body weight	Underweight	0 (0)	15 (13.6)	**0.001**
	Normal weight	21 (22.6)	27 (24.5)	
	Overweight	37 (39.8)	23 (20.9)	
	Obese	29 (31.2)	36 (32.7)	
	Morbid obesity	6 (6.5)	9 (8.2)	

Data are presented as numbers and percentages. A Chi-square test was applied. Bold values indicate statistical significance at *p* < 0.05.

**Table 2:** Genotype and allele frequencies of *SENCR* rs12420823*C/T.

Variant	Total	Controls	Patients	*p*-Value	Crude OR (95%CI)
Total number	203	93	110		
Allele frequency					
C	194 (47.8%)	105 (56%)	89 (40.4%)	**<0.001**	1
T	212 (52.3%)	81 (44%)	131 (59.6%)		**1.91 (1.28–2.83)**
Genotype frequency					
C/C	37 (18.2%)	26 (28.0%)	11 (10.0%)	**0.001**	1
C/T	120 (59.1%)	53 (57.0%)	67 (60.9%)		1.82 (0.88–3.70)
T/T	46 (22.7%)	14 (15.0%)	32 (29.1%)		**5.26 (2.08–14.3)**

Data are presented as frequency (percentage). A two-sided Chi-square test was used. Bold values indicate statistical significance at *p* < 0.05. OR: Odds ratio; CI: confidence interval.

**Table 3:** Genetic association model for cancer risk.

Model	Genotype	Control	Patients	Adjusted OR (95% CI) #	*p*-Value	AIC	Adjusted OR (95% CI) *	*p*-Value	AIC
Codominant	C/C	26 (28%)	11 (10%)	1	**0.001**	272.6	1	**0.001**	233.7
	C/T	53 (57%)	67 (60.9%)	1.61 (0.78–3.45)			1.85 (0.72–4.76)		
	T/T	14 (15.1%)	32 (29.1%)	**5.26 (2.04–14.29)**			**8.33 (2.44–25.0)**		
Dominant	C/C	26 (28%)	11 (10%)	1	**0.027**	272.3	1	**0.019**	233.3
	T/T-C/T	67 (72%)	99 (90%)	**3.70 (1.72–8.33)**			**5.56 (2.0–14.3)**		
Recessive	C/T-C/C	79 (85%)	78 (70.9%)	1	**<0.001**	279.5	1	**<0.001**	240
	T/T	14 (15.1%)	32 (29.1%)	**2.17 (1.08–4.55)**			**2.86 (1.15–7.14)**		
Over-dominant	C/C-T/T	40 (43%)	43 (39.1%)	1	0.36	283.6	1	0.44	244.9
	C/T	53 (57%)	67 (60.9%)	1.31 (0.73–2.53)			1.34 (0.64–2.83)		

Data are presented as frequency (percentage). OR: odds ratio; CI: confidence interval; AIC: Akaike information criterion. The logistic regression model was employed. # Adjusted by age. * Adjusted by age and other risk factors such as the family history of cancer, oral contraceptive pills or hormonal replacement therapy, early menarche, nullipara, late first gravida, late menopause, no breastfeeding, night light exposure, sedentary lifestyle, smoking, and obesity. Bold values indicate statistical significance at *p* < 0.05.

**Table 4:** Association of *SENCR* rs12420823*C/T variant with the histopathological types of BC in the studied patients.

Histopathological Type	C/C	C/T	T/T	*p*-Value
Duct carcinoma	6 (54.5)	24 (35.8)	12 (37.5)	0.68
Lobular carcinoma	2 (18.2)	16 (23.9)	10 (31.3)	
Invasive medullary carcinoma	2 (18.2)	10 (14.9)	2 (6.3)	
Mucinous carcinoma	0 (0)	9 (13.4)	2 (6.3)	
Tubular carcinoma	0 (0)	5 (7.5)	3 (9.4)	
Metaplastic carcinoma	1 (9.1)	3 (4.5)	3 (9.4)	

Data are presented as frequency (percentage). A two-sided Chi-square test was used. Statistical significance was set at *p* < 0.05.

**Table 5:** Association of *SENCR* rs12420823*C/T variant with demographic features and risk factors.

Characteristics	Levels	C/C-C/T	T/T	*p*-Value
Demographics				
Age	Adolescents (≤21 years)	63 (80.8)	25 (78.1)	0.79
	Adults (>21 years)	15 (19.2)	7 (21.9)	
Residency	Urban	31 (39.7)	12 (37.5)	0.82
	Rural	47 (60.3)	20 (62.5)	
Marital status	Divorced	16 (20.5)	3 (9.4)	0.026
	Married	34 (43.6)	23 (71.9)	
	Single	28 (35.9)	6 (18.8)	
Occupation	Housewife	56 (71.8)	24 (75)	0.81
	Worker	22 (28.2)	8 (25)	
Menopausal status	Pre-menopause	58 (74.4)	21 (65.6)	0.31
	Post-menopause	20 (25.6)	11 (34.4)	
Risk factors				
Family history of cancer	Negative	55 (70.5)	23 (71.9)	0.88
	Positive	23 (29.5)	9 (28.1)	
Oral contraceptive pills	Negative	60 (76.9)	29 (90.6)	0.15
	Positive	18 (23.1)	3 (9.4)	
Early menarche	Negative	28 (35.9)	10 (31.3)	0.82
	Positive	50 (64.1)	22 (68.8)	
Nullipara	Negative	66 (84.6)	28 (87.5)	0.77
	Positive	12 (15.4)	4 (12.5)	
Late first gravida	Negative	75 (96.2)	31 (96.9)	0.85
	Positive	3 (3.8)	1 (3.1)	
Late menopause	Negative	69 (88.5)	29 (90.6)	0.74
	Positive	9 (11.5)	3 (9.4)	
No breastfeeding	Negative	63 (80.8)	27 (84.4)	0.78
	Positive	15 (19.2)	5 (15.6)	
Night light exposure	Negative	70 (89.7)	28 (87.5)	0.74
	Positive	8 (10.3)	4 (12.5)	
Sedentary lifestyle	Negative	9 (11.5)	2 (6.3)	0.50
	Positive	69 (88.5)	30 (93.8)	
Smoking	Negative	69 (88.5)	29 (90.6)	0.71
	Positive	9 (11.5)	3 (9.4)	
Obesity	Negative	30 (38.5)	12 (37.5)	0.92
	Positive	48 (61.5)	20 (62.5)	

Data are presented as frequency (percentage). A two-sided Chi-square test was used. Statistical significance was set at *p* < 0.05.

**Table 6:** Association of *SENCR* gene variant with the clinicopathological data of the patient group.

Characteristics	Levels	C/C-C/T	T/T	*p*-Value	OR (95%CI)
Clinical presentation					
Mastalgia	Positive	27 (34.6)	8 (25)	0.37	0.63 (0.25–1.59)
Breast mass	Positive	64 (82.1)	28 (87.5)	0.58	1.53 (0.46–5.07)
Skin changes	Positive	12 (15.4)	5 (15.6)	0.97	1.02 (0.33–3.17)
Nipple changes	Positive	16 (20.5)	2 (6.3)	0.08	0.26 (0.06–1.2)
Axillary pain	Positive	5 (6.4)	2 (6.3)	0.97	0.97 (0.18–5.3)
Axillary mass	Positive	5 (6.4)	2 (6.3)	0.97	0.97 (0.18–5.3)
Pathological data					
Focality	Unifocal	62 (79.5)	22 (68.8)	0.32	Reference
	Multifocal	16 (20.5)	10 (31.3)		1.76 (0.7–4.45)
Pathological grade	Grade 2	62 (79.5)	26 (81.3)	0.83	Reference
	Grade 3	16 (20.5)	6 (18.8)		0.89 (0.31–2.54)
Tumor stage	T2 stage	38 (48.7)	13 (40.6)	0.52	Reference
	T3/4 stages	40 (51.3)	19 (59.4)		1.39 (0.6–3.2)
Nodal stage	Negative infiltration	21 (26.9)	8 (25)	0.83	Reference
	Positive infiltration	57 (73.1)	24 (75)		1.11 (0.43–2.84)
NPI	Good	37 (47.4)	17 (53.1)	0.67	0.8 (0.35–1.82)
	Poor	41 (52.6)	15 (46.9)		
ESMO	Low risk	29 (37.2)	11 (34.4)	0.83	1.13 (0.48–2.68)
	High risk	49 (62.8)	21 (65.6)		
Receptor status					
ER/PR	Positive	47 (60.3)	12 (37.5)	**0.036**	0.4 (0.17–0.92)
HER2^+^	Positive	9 (11.5)	8 (25)	0.08	2.56 (0.89–7.37)
TNBC	Positive	28 (35.9)	17 (53.1)	0.13	2.02 (0.88–4.66)
IHPI	Good	47 (60.3)	12 (37.5)	0.08	Reference
	Moderate	28 (35.9)	17 (53.1)		0.84 (0.35–2.02)
	Poor	3 (3.8)	3 (9.4)		2.33 (0.41–13.2)
Clinical outcomes					
Recurrence	Negative	42 (53.8)	8 (25)	**0.006**	3.5 (1.4–8.74)
	Positive	36 (46.2)	24 (75)		
Survival	Prolonged > 12 months	55 (70.5)	13 (40.6)	**0.005**	3.5 (1.49–8.33)
	Short ≤ 12 months	23 (29.5)	19 (59.4)		

Data are presented as frequency (percentage). A two-sided Chi-square test was used. OR: odds ratio; CI: confidence interval. NPI: Nottingham Prognostic Index, calculated as (0.2 × tumor size in cm) + tumor grade (1–3) + lymph node stage (1–3, according to stages A–C); ESMO: European Society of Medical Oncology; ER/PR: estrogen and progesterone receptors; HER^2+^: HER2/neu receptor; TNBC: triple-negative breast cancer; IHPI: Immunohistochemical Prognostic Index estimated based on the three receptor statuses (HER2, ER, and PR). Bold values indicate statistical significance at *p* < 0.05.

**Table 7:** Linkage disequilibrium (LD) and impact of the studied *SENCR* rs12420823 variant on chromosome 11 with other detected variants (r^2^ ≥ 0.8) on the same chromosome.

Position (hg38)	LD (r²)	LD (D′)	Variant	Ref	Alt	AFR Freq	AMR Freq	ASN Freq	EUR Freq	Promoter Histone Marks	Enhancer Histone Marks	Proteins Bound	Motifs Changed	dbSNP Func Annot
128693497	1	1	**rs12420823**	C	T	0.51	0.40	0.20	0.47	21 tissues	4 tissues	CTCF, CJUN		intronic
128693518	1	1	rs12420835	C	A	0.39	0.38	0.19	0.47	22 tissues	4 tissues	CTCF, CJUN	9 altered motifs	intronic

“Position, hg38: human genome release number 38, LD: linkage disequilibrium, Ref: reference allele, Alt: alternative allele, AFR: African, AMR: American, ASN: Asian, EUR: European, freq: frequency, dbSNP func annot: Database of single nucleotide polymorphism functional annotation”. CTCF: transcriptional repressor, also known as “11-zinc finger protein” or “CCCTC-binding factor”; CJUN: transcription factor Jun. The bold SNP is the specified variant in this study. Data source: HaploReg v 4.1. (https://pubs.broadinstitute.org/mammals/haploreg/haploreg.php) (accessed on 20 September 2022).

## Data Availability

All generated data in this study are included in the submitted article.
